# Addressing Racism and Its Deeply Entrenched Dynamics: A 21st Century Imperative

**DOI:** 10.1089/heq.2022.29018.gcc

**Published:** 2023-01-13

**Authors:** Gail C. Christopher

**Affiliations:** Executive Director, National Collaborative for Health Equity, Washington, District of Columbia, USA.

Throughout the world, extreme nationalism, racism, anti-Semitism, and other forms of ethnic and religious bias are sustained by an antiquated notion that the human family can be divided and ranked based on physical characteristics and ascribed traits. This belief in a hierarchy of human value fuels racism in America, dividing our population and providing platforms for hatred and attacks on our democracy. The foundational fallacy of human hierarchy continues to spur many inequities both consciously and unconsciously through systems and individual acts. As this ill-conceived belief ossifies, divisiveness hardens among communities, creating barriers to unifying our population into a critical mass that is committed to equity in our communities, in our health care, in education, in employment, and all aspects of our society.

We must focus our energy, resources, and discourse on uprooting and eliminating the false ideology of a hierarchy of human value and seed what must be valued most: our common humanity. When our beliefs and understandings shift, our capacity for empathetic relatedness, civility, and progress toward achieving common interests will increase. Beliefs, particularly deeply held and hardened beliefs, have a way of influencing, if not shaping, our lives. It is past time to overturn this belief in a hierarchy of human value and address, and redress, its legacy of discriminatory policies and practices that create unhealthy outcomes for people of color.

Research reveals that the inequities caused by racism cost our nation almost $2 trillion annually in lost purchasing power, reduced job opportunities, and diminished productivity.^1^ What is also clear is that the conscious and unconscious belief in a racial hierarchy fuels the reluctance of political leaders and policymakers to acknowledge the inequities and devote adequate resources to addressing them. Our democracy, similar to others around the world, is based upon full human engagement and action on shared interests for the population. Clearly, to move forward, this nation must heal the wounds of our past and learn to work together with civility, and indeed, with love. We must build the individual and collective capacity to “see ourselves in the face of the other.”

We know the stress of racism predisposes the body to chronic illness. Our inability as individuals and as a society to value all human beings equally is making us sick, literally. Even more broadly speaking, the incapacity to value all human beings equally keeps us from experiencing optimal well-being and happiness. Our hearts and brains are designed to resonate within harmonious relationships. The opposite—fear and anxiety, separation, alienation, and hate—induces stress and distress. Distress causes a cascade of illness related changes within our very cells in our physical bodies and within our body politic.

Researchers estimate that 265 people die every day from racial health disparities in the United States. But it is not just people of color who suffer and die prematurely.^2^ The U.S. population, as a whole, lives shorter lives and has poorer health than our peer nations. Our residual belief in a false taxonomy and hierarchy of value is a major contributing factor to our poor health outcomes. Distress responses related directly and indirectly to racial fear, anxiety, and to its attendant social conditions contribute to hypertension and cardiovascular disease, glucose intolerance, insulin resistance, and diabetes and its precursor metabolic syndrome.^3^

The National Collaborative for Health Equity (NCHE) is committed to addressing the deep racial and ethnic inequities that adversely affect the health of men, women and children living in communities of color. Our mission is to dismantle racism through three intervention areas: supporting local leaders, providing and facilitating data that help create authentic narratives, and supporting grassroot efforts that are engaging racism on the ground at the local level.

With the support of our funders, NCHE provides institutions and leaders from historically marginalized and excluded communities with tools to improve the social, economic, and environmental conditions that shape health. These tools include leadership development, policy analysis, data mining and analysis, and community organizing and mobilization to address a range of issues that are the ultimate determinants of health. We are committed to addressing the deep racial and ethnic health inequities that persist largely due to structural and institutional racism and exclusion. Furthermore, NCHE places a priority on supporting and connecting leaders in communities that are creating the change America so badly needs.

Since the first European settlers arrived on our shores centuries ago, American culture has placed the relative worth of White people above all others, and at times violently enforced this. The adopted and embedded mindset and belief have restricted the quality of life for people of color, whereas limiting opportunities for success and preventing the realization of the full potential of our democracy. America's history includes enslaving people, committing genocide among the Indigenous population, and embracing centuries of institutionalized racism.

Yet, unlike other countries that have endured war, sectarian, or racial strife, the United States has never established a comprehensive Truth and Reconciliation Commission effort to heal divisions and bring equal opportunities to all communities. As a result of the unequal conditions caused by structural racism and discriminatory policies and practices, the nation experiences a significant wealth gap between White people and families of color, persistent residential segregation, unequal access to quality health care and affordable housing, achievement gaps in education, and discrimination in hiring practices.

In fact, the 2021 Survey of Income and Program Participation found that the median White family has 12 times the amount of wealth of the median Black family, a disparity translating to $18,430 of median net worth for Black households compared with $217,500 in median net worth for White households.^4^ Truly, America has two societies, one predominantly White and privileged, and another Black and Brown where daily struggles for health care, food, housing, jobs, and other social determinants of health exist.

In 2016, under the guidance of Dr. Gail Christopher, the W.K. Kellogg Foundation launched Truth, Racial Healing & Transformation (TRHT), a national and community-based effort to engage communities, organizations, and individuals from multiple sectors across the United States in racial healing and addressing present-day inequities linked to historic and contemporary beliefs in a hierarchy of human value. A year-long design process was informed by 176 community and civic leaders, scholars, and practitioners. The TRHT framework seeks to jettison the belief in a hierarchy of human value by centering transformative approaches to community-based healing and supporting the pursuit of actionable change. It has been implemented in an array of communities, including >70 college and university campuses.

At NCHE, our initiatives and programs are all grounded by the TRHT framework, which include five pillars that guide communities and organizations in implementing TRHT-driven initiatives. *Narrative Change* examines how to create a more complete and accurate narrative that will help people understand how racial hierarchy has been embedded in our society from the beginning. *Racial Healing and Relationship-Building* focuses on ways for all of us to heal from the wounds of the past and build mutually respectful relationships across racial and ethnic lines that honor and value each person's humanity. *Separation* examines and finds ways to address segregation, colonization, and concentrated poverty in neighborhoods. *Law* reviews discriminatory civil, criminal, and public policies, and seeks solutions that will produce a more just application of the law. *Economy* studies structured inequality and barriers to economic opportunities and developing solutions that will create a more equitable society.

Today, NCHE is proud to present our inaugural group of NCHE Senior Scholars, five distinguished academic and social justice leaders, who have each written comprehensive articles that align with the TRHT pillars. Their knowledge and insights will be shared with the NCHE network through publications, webinars, and this special roundtable event with Health Equity, a peer-reviewed journal that is NCHE's official publication partner. We will all benefit from having access to the expertise and insights that these scholars can provide as NCHE continues its mission to advance health equity in communities of color.

The Senior Scholars, who you will learn more about shortly, are as follows:

- Charmaine Royal, PhD, MS, is the Robert O. Keohane Professor of African & African American Studies, Biology, Global Health, and Family Medicine & Community Health at Duke University. She directs the Duke Center on Genomics, Race, Identity, Difference and the Duke Center for TRHT. In her article, Royal examined the narrative change pillar, and concluded there must be “a shift in our approach” if America is to truly address the systemic racism embedded in our society. She writes that approaches with “focuses almost exclusively on the consequences of racism and racialization (must shift) to one that devotes adequate attention to both the consequences and the root cause (the underlying belief system) of these inhumane processes. Just as science and society cooperatively invented and perpetuated the illusion of race, they must join forces to undo this deed, generating and circulating counter-narratives that will help inform and transform America and the world. Race is inextricably associated not only with assumed innate differences between human populations, but also a hierarchy of difference wherein one population is deemed superior to another. It is this twofold formula of racism, not actual biological difference, that shaped ideas about race and racial classifications in humans.”
- Lisa Sockabasin, MS, is a citizen of the Passamaquoddy Tribe at Motahkomikuk with extensive experience in Tribal, State, and Federal governments, nonprofits, and philanthropic organizations. In her capacity as the Co-CEO of Wabanaki Public Health and Wellness (WPHW), Sockabasin collaborates with tribal leadership, the WPHW team, and federal and philanthropic partners to address systemic inequities experienced by Wabanaki communities in Maine and to develop and implement culturally based programs that respond to the needs of our communities. Focused on racial healing and relationships, she writes eloquently about the state of our society and how to get it back on track: “The absence of indigenous ways of being, knowing, and connecting has immense impact on our nation's ability to live in relationship with this land and with each other. The divisiveness unyielding in our society, easily found with a quick scroll through social media or a click on the television, we are all painfully aware. Our land, also experiencing the loss of the indigenous worldviews of holding the land with care and reciprocity. The lack of indigenous worldviews in mainstream society is a society that lacks connection and respect for all, a society that values individualism rather than collectivism. Indigenous values express the importance of respecting the sovereignty of the land and her people. Viewing the land as a sacred partner, rather than an object of possession and control. As a partner, the land and her people serve each other, providing nourishment collectively. The loss of indigenous voice and leadership across America, has created a great imbalance. We have become a nation of people believing in invisibility and the retelling of history, a place where divisiveness is dominant and seeing truth is too painful. While indigenous people have paid the highest price, all others who share this land, must understand the devastation will be and is being shared with each of us.”
- Mindy Thompson Fullilove, MD, LFAPA, Hon AIA, is a social psychiatrist and professor of urban policy and health at The New School. Since 1986, she has conducted research on AIDS and other epidemics of poor communities. With a special interest in the relationship between the collapse of communities and decline in health. Fullilove examines the separation pillar and its impact on health. In the reality of the workings of ecological systems, “separation” is an illusion that, paradoxically, binds the parts more tightly together. Her article explores three themes that are important today: (1) the paradox of apartheid that “separation” binds the parts more firmly together; (2) the ongoing “redlining system,” which continues to ensnare us in a geography of apartheid; and (3) the “new racism,” which attacks progress with the use of such farfetched ideas as the “replacement theory.” “It is impossible to make progress if we aren't actually making progress,” she writes. “The real issues like housing, wages, and climate change must be front and center all the time. Racism was invented to divide us and to hide the scam that's going on. Let's not forget what's behind the curtain.”
- Alan Jenkins, JD, MA, is a Professor of Practice at Harvard Law School where he teaches courses on Race and the Law, Communication, and Social Justice. Jenkins was president and cofounder of The Opportunity Agenda, a social justice communication laboratory dedicated to the idea that America can and should be a place where everyone enjoys full and equal opportunity. In his article, Jenkins recounts the turbulent relationship between law and equity in our nation, discusses the elements that can lead to major progress through law, and recommends specific steps that different actors can take to move an equity and opportunity agenda forward. “History and experience teach us that our Constitution and laws can be instruments of racial discrimination and oppression as well as tools for advancing freedom and equality,” he writes. “The substance of our laws matters, and there is much to be learned from innovative policies and legal strategies around the country. In addition to laws promoting health equity and racial justice at the state, tribal, and local levels, a new federal Executive Order has the potential to drive major positive change if fully and properly implemented. Just as important, however, is linking legal advocacy with dynamic social movements, shrewd communication strategies, and courageous civic leadership that insists on transformative change. In this article, I briefly recount the turbulent relationship between law and equity in our nation; discuss the elements that can lead to major progress through law; and recommend specific steps that different actors can take to move an equity and opportunity agenda forward.”
- Algernon Austin, PhD, is the Director for Race and Economic Justice at the Center for Economic and Policy Research. He has conducted research and writing on issues of race and racial inequality for >20 years. His primary focus has been on the intersection of race and the economy and his TRHT focus is the economy. In his article, Austin writes that racist ideas and practices help to structure American society by being in dialogue with the economy of the society. The dominant racist ideologies of a society economically reliant on racial slavery will justify and support racial slavery. He notes that the economy of American society no longer rests on racial slavery, so there are no longer ideologies justifying the importation of Africans. A key part of racism is the creation of myths to justify economic hierarchies. Racial slavery was a profitable institution for slaveholders. When slavery was challenged, the historian Peter Kolchin reports, “Southern spokesmen responded with elaborate arguments in defense of slavery, including pseudoscientific demonstrations that blacks were unfit for freedom, reminders that the nation's economic well-being depended on slave labor, assertions that the Bible itself sanctioned the enslavement of the ‘sons of Ham,’ and claims that slavery produced a more humane and harmonious social order than the exploitative free-labor system.” The desire to profit from racial slavery encouraged the development and dispersal of ideas to justify it. Looking at the broad history of racist ideologies in America, the historian Mia Bay notes that one sees “ever-changing intellectual rationalizations” to justify racial economic hierarchy.

This publication should be of interest and value to executive leaders in all sectors across the country. It provides the content of the roundtable conversation between the Senior Scholars as well as their comprehensive articles that align with each pillar of the TRHT framework. We believe that 21st century leadership requires addressing the unresolved issues of racism and it is deeply entrenched dynamics. We encourage you to read and share this publication.

Sincerely,

Gail C. Christopher


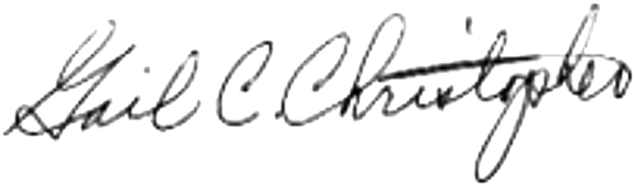


## Abbreviations Used

NCHE: National Collaborative for Health Equity
TRHT: Truth, Racial Healing & Transformation
WPHW: Wabanaki Public Health and Wellness
